# Adenovirus with DNA Packaging Gene Mutations Increased Virus Release

**DOI:** 10.3390/v8120333

**Published:** 2016-12-20

**Authors:** Stephen L. Wechman, Xiao-Mei Rao, Kelly M. McMasters, Heshan Sam Zhou

**Affiliations:** 1 Department of Pharmacology and Toxicology, University of Louisville School of Medicine, Louisville, KY 40202, USA; slwech01@louisville.edu; 2 Department of Surgery, University of Louisville School of Medicine, Louisville, KY 40202, USA; x0rao001@louisville.edu; 3 James Graham Brown Cancer Center, University of Louisville School of Medicine, Louisville, KY 40202, USA; 4 Department of Microbiology and Immunology, University of Louisville School of Medicine, Louisville, KY 40202, USA

**Keywords:** lung cancer, adenovirus, *E1b*, UV-irradiation, genomics

## Abstract

Adenoviruses (Ads) have been extensively manipulated for the development of cancer selective replication, leading to cancer cell death or oncolysis. Clinical studies using *E1*-modified oncolytic Ads have shown that this therapeutic platform was safe, but with limited efficacy, indicating the necessity of targeting other viral genes for manipulation. To improve the therapeutic efficacy of oncolytic Ads, we treated the entire Ad genome repeatedly with UV-light and have isolated AdUV which efficiently lyses cancer cells as reported previously (Wechman, S. L. et al. Development of an Oncolytic Adenovirus with Enhanced Spread Ability through Repeated UV Irradiation and Cancer Selection. *Viruses* **2016**, *8*, 6). In this report, we show that no mutations were observed in the early genes (*E1* or *E4*) of AdUV while several mutations were observed within the Ad late genes which have structural or viral DNA packaging functions. This study also reported the increased release of AdUV from cancer cells. In this study, we found that AdUV inhibits tumor growth following intratumoral injection. These results indicate the potentially significant role of the viral late genes, in particular the DNA packaging genes, to enhance Ad oncolysis.

## 1. Introduction

Human adenoviruses (Ads) which have been modified for cancer selective replication, known as oncolytic virotherapeutics, comprise a very promising cancer therapeutic platform for the treatment of solid tumors [1]. Cancer selective Ads have very minor side effects including negligible flu-like symptoms [2]. There are no long-term complications associated with Ad treatment as these viruses do not have a latent phase infection nor do their genomes integrate into cellular chromosomes [3]. *E1b*-deleted Ads, such as *dl*1520 and H101, have been shown to replicate within tumors while only inducing fever in a dose-dependent manner beyond those symptoms observed from cancer chemotherapy [3,4,5]. The therapeutic effects of oncolytic Ads are initiated from a small number of infected cancer cells from which progeny viruses spread to infect adjacent cancer cells within tumors [6,7]. However, preclinical and clinical studies have suggested that Ad spread is restricted in large solid tumors, limiting their therapeutic efficacy [8,9,10,11,12].

The Ad genome, composed of linear double-stranded DNA of approximately 36 kilobases (kb), is divided into early (*E*) and late (*L*) genes based upon the expression of these genes across time [3,13]. The L genes encode structural proteins which package the viral DNA into the Ad virion during the final stages of replication. The E genes include *E1a*, *E1b*, *E2*, *E3* and *E4*. The development of oncolytic Ads has primarily focused on the genetic manipulation of *E1a* and *E1b*. The proteins encoded by *E1a* are produced immediately after infection to modulate the cell cycle, recruit cellular proteins, and regulate the expression of cellular and viral genes [14]. The Ad *E1b* gene encodes two major polypeptides of 55,000 kDa (55K) and 19,000 kDa (19K). The expression of *E1b*55K, but not *E1b*19K, has been shown to cooperate with *E1a* to transform rodent cells [15,16]. Both E1B55K and E1B19K proteins have been shown to protect infected cells from E1A-induced stabilization of p53 and apoptosis [17]. E1B55K also enhances viral *E1a* expression and is involved in the induction of cyclin E required for Ads to efficiently replicate [9,18,19,20,21,22,23]. The Ad E1B19K protein is a putative B-cell lymphoma 2 (Bcl-2) functional homolog [24,25,26] which prevents E1A-induced apoptosis by inhibiting the pro-apoptotic proteins Bak and Bax [27]. The pleiotropic effects of E1B19K, E1B55K and E1A peptides acting simultaneously are therefore thought to create an intracellular environment which maximizes Ad replication.

Two major strategies have been applied for the development of oncolytic Ads. One is to delete or attenuate viral genes, such as *E1b*, which are not essential for virus replication in cancer cells. Ad *dl*1520 (ONYX-015), for example, is a gene-attenuated oncolytic virus with an 827-bp deletion and a nonsense point mutation in *E1b* that generates a premature stop codon to prevent the complete translation of the E1B55K protein [15,28,29]. Ad *dl*1520 has shown promising oncolytic efficacy in preclinical and clinical trials. Several hundred cancer patients were treated with *dl*1520 via various routes of injection in phase I and phase II clinical trials [30]. However, during a phase III clinical trial, the combination of *dl*1520 and chemotherapy treatment was suspended due to limited therapeutic efficacy [31]. Another strategy to develop cancer selective Ads is to restrict the expression of essential viral regulatory genes, such as *E1a*, using cancer selective promoters [7,32]. Oncolytic Ads with *E1a* controlled by cancer-selective promoters, such as OBP-301 (Telomelysin®) driven by human telomerase reverse transcriptase (hTERT) promoter [33] and CV706 driven by prostate-specific antigen (PSA) promoter [34], have progressed to human clinical trials. These viruses were shown to be safe, but without producing clinically relevant therapeutic responses. We recently developed Ad-cycE, in which *E1a* is regulated by the human cyclin E promoter [23,35]. Cyclin E overexpression has been observed in lung, liver, gastrointestinal, glioma/blastoma, bone, and breast cancers [36]. We have previously shown that Ad infection stimulated the activity of the cyclin E promoter [19,20], therefore, the inclusion of the cyclin E promoter to control *E1a* augmented the oncolytic efficacy of Ads [35,37].

Although Ads with *E1b* deletions or *E1a* regulated by cancer selective promoters have achieved some success in human clinical trials, the efficacy of oncolytic Ad virotherapy overall has been disappointing [8,29,31,38,39]. Oncolytic Ads were unable to repress the growth of large tumors in vivo [8] and in patients [29,39]. Therefore, restricted virus spread in large tumors represents a major hurdle limiting the efficacy of oncolytic virotherapy [40]. More than a decade of research has suggested that genetic manipulation of only the Ad early genes is not sufficient to overcome this hurdle. Therefore, it is necessary to explore the utility of modifying other Ad genes for the development of more effective oncolytic Ads.

We have previously developed AdUV, which was isolated from a pool of the adenovirus serotype 5 *dl*309 (Ad5) viral particles which were repeatedly treated with UV type-C irradiation [41]. AdUV was shown to lyse cancer cells very effectively and formed large viral plaques on cancer cell monolayers. In this study, we extended our previous studies to identify AdUV genome mutations. No mutations were observed in the early genes (*E1*, *E4*) in AdUV, but several mutations were found within the late genes encoding Ad structural proteins involved in viral DNA packaging. AdUV was shown to effectively lyse cancer cells while safely and efficiently suppressing A549 tumor xenograft growth in nude mice.

## 2. Materials and Methods

### 2.1. Cell Lines and Culture Conditions

HEK293 (ATCC no. CRL-1573) human embryonic kidney, MRC5 (ATCC no. CCL-171) human non-cancerous lung fibroblast, HBEC (ATCC no. CRL-4051) human non-cancerous human bronchial epithelial cells, A549 (ATCC no. CCL-185) human lung carcinoma and Saos-2 (ATCC no. HTB-85) human osteosarcoma cells were all purchased from the ATCC (Rockville, MD, USA). A549, MRC5 and HEK293 cells were maintained in Dulbecco's Modified Eagle's Medium (DMEM). Saos-2 cells were maintained in McCoy’s 5A media. DMEM and McCoy’s 5A media were supplemented with 10% fetal bovine serum, L-glutamine, and penicillin/streptomycin (100 U/mL). HBEC cells were cultured in airway epithelial cell basal medium (ATCC PCS-300-030) supplemented with bronchial epithelial cell growth kit (ATCC PCS-300-040). All other cell culture reagents were obtained from VWR (VWR, Radnor, PA, USA). All cells were cultured and maintained in humidified 5% CO_2_ incubators at 37 °C.

### 2.2. Adenoviral Vectors

Ad5 *dl*309 is *E1*-wildtype virus [42] and was used as a non-selective Ad control. This virus also contains deletions in *E3*, preventing the expression of *E3B* 10.4K, 14.6K, and 14.7K genes, but not the *E3A* glycoprotein (gp) 19K gene [42]. Adhz60 is an *E1b*-deleted vector similar to *dl*1520 and H101 and was used as a cancer selective control in these studies [43]. AdGFP is *E1* (*E1a* and *E1b*) deleted, with green fluorescent protein (GFP) expression driven by the cytomegalovirus (CMV) promoter [35]. AdGFP, as a negative control, does not replicate nor induce cytopathic effects (CPE) [9,23]. All Ads used in this study express *E1* and *E4* unless otherwise specified, and are based upon Ad5 backbone sequences (GENEID# AC_000008.1).

To select Ads with increased spread, Ad5 *dl*309 was treated with UV irradiation for 5 min, inactivating approximately 90% of virus particles. The UV-light source was a germicidal lamp, USEG30T3 (Sylvania, Danvers, MA, USA), fitted with a 30 watt G30T8 UV-light bulb (Philips, Amsterdam, The Netherlands), which produces UV type-C irradiation. UV irradiation was measured with a model 25X UVX radiometer (Fisher Scientific, Pittsburgh, PA, USA). Using these conditions, 213 μW/cm^2^ UV type-C was produced, generating a total UV dose of 639 J/m^2^ after 5 min exposure. Irradiated viruses were then used to infect Saos-2 cancer cells, in which Ads cannot replicate as well as in HEK293 and A549 cells [20]. Four hours (h) after infection, virus particles in the medium were removed by washing the cells with fresh media. Medium from infected monolayer cultures was collected at the first sign of CPE, generally at 24 h after infection, to harvest viruses that might replicate more efficiently in and release from the infected cancer cells. The harvested viruses were then amplified in HEK293 cells before subsequent cycles of UV-light treatment. These treatments were repeated for 72 cycles. AdUV was then isolated from a mixture of viruses by plaque purification and selection for those Ads forming large plaques on Saos-2 cells as previously described [41].

### 2.3. Virus Titration and Release

Virus titer was determined using the median tissue culture infective dose (TCID50) method [8,22,44]. HEK293 cells were seeded overnight onto 96-well plates at a density of 1 × 10^3^ cells per well and infected with virus samples serially diluted tenfold. The presence or absence of CPE in HEK293 cells was recorded, after a minimum of seven days, to calculate the virus titer.

To determine the release kinetics of AdUV, A549 and Saos-2 cells were seeded onto 12-well plates at a density of 1 × 10^5^ and 1.5 × 10^5^ cells per well, respectively. A549 cells were then infected with Ad5, Adhz60 or AdUV at a multiplicity of infection (MOI) of 1. The cells and cell culture samples were then collected at 6, 24, 36, 48, 72, 96 and 120 h post-infection. The cells were separated from the cell culture media and discarded from each sample following centrifugation at 2000 RPM (350 RCF) at 4 °C for 5 min using a micromax RF refrigerated microcentrifuge equipped with an IEC 851 rotor (Thermo Fisher Scientific, Waltham, MA, USA). Cell free media samples were titered to detect the concentrations of Ads released.

### 2.4. Cytotoxicity Assay

Cells were seeded at a density of 3 × 10^4^ (A549) or 4.5 × 10^4^ (Saos-2) cells per well onto 24-well plates and allowed to adhere overnight. Cytotoxicity was assessed by crystal violet staining after five days [45]. Suspended cells were aspirated and the adherent cells were then fixed via incubation with 3.7% formaldehyde for 25 min at room temperature (RT). Excess formaldehyde was washed away using phosphate-buffered saline (PBS). Cells were then stained using 1% crystal violet for 30 min at RT. Excess crystal violet was washed away with water. These crystal violet stained wells were then scanned using an HP Scanjet 4070 scanner (HP, Palo Alto, CA, USA). The remaining crystal violet was then solubilized with a 2% sodium dodecyl sulfate (SDS) solution and the sample absorbances were measured at 590 nm using a Synergy HT Multi-Mode Microplate Reader (Bio-Tek, Winooski, VT, USA). The absorbance (OD) values were then normalized to mock-treated cells converting each sample OD into the percent (%) cell viability by the formula, cell viability % = (OD of treated cells/OD of mock-treated cells) × 100.

### 2.5. DNA Sequencing

DNA sequencing was performed at the University of Louisville (Louisville, KY, USA) KBRIN bioinformatics core facility using Ion PGM Sequencing 200 kit v2 and Ion PGM system. An Ion 314 v2 Chip was used for loading the enriched Ion One Touch 200 ISP samples. Open source integrated genomics viewer (IGV) software was downloaded to view these DNA sequencing data. Mutations with low DNA sequencing error rates were listed in Table 1.

### 2.6. Lung Cancer Xenograft

Female athymic nude mice (NCr-nu/nu) were obtained from the National Cancer Institute (Bethesda, MD, USA). Tumors were formed following the subcutaneous injection (SC) of 5 × 10^6^ A549 lung cancer cells into the right flanks of NCr-nu/nu mice at six weeks of age. Ads were titrated on HEK293 cells using the plaque assay to determine the plaque forming units (PFU) for AdUV and AdGFP prior to intratumoral injection as described previously [46,47]. Once palpable tumors were established and reached 30 mm^3^, mice were randomized into two treatment groups to receive 5 × 10^8^ PFU of AdGFP (*n* = 8) or AdUV (*n* = 7) in 50 µL PBS every three days for a total of four treatments (2 × 10^9^ PFU) from days 6 to 15. Tumor volumes were determined by externally measuring these tumors in two dimensions with a caliper and calculated based upon the following equation, V = (L × W^2^)/2, where L is length and W is the width of the tumor as described previously [9,23]. Eight mice were treated with AdGFP and seven mice were treated with AdUV. Animal experiments were performed according to the institutional guidelines approved by the University of Louisville Institutional Animal Care and Use Committee (IACUC, protocol # 11079, Louisville, KY, USA).

### 2.7. Immunohistochemistry

Tumors were harvested seven days following the fourth treatment and embedded in optimal cutting compound (OTC; Sakura Finetek, Torrance, CA, USA), and stored at −20 °C. Briefly, slides were air-dried and fixed in ice-cold methanol at 4 °C for 20 min. The endogenous tissue peroxidase activity was blocked by in a 0.3% hydrogen peroxide 3% methanol solution for 30 min according to the Vectastain ABC protocol (Vector Laboratories, Burlingame, CA, USA). These tissues were then blocked in SuperBlock (Pierce Biotechnology, Rockford, IL, USA) for 45 min at RT. Sections were incubated with goat-anti-adenovirus polyclonal antibody (Millipore, Billerica, MA, USA), diluted 1:800, overnight at 4 °C. The signals were amplified by a biotinylated anti-goat IgG diluted 1:200 in conjunction with Vectastain avidin-biotin complex method kit (Vector Laboratories, Burlingame, CA, USA). Visualization was achieved using 3,3-diaminobenzidine-tetrahydrochloride (DAB) for 5 min at RT; the reaction was stopped by dipping the slide in water (ImmPACT DAB peroxidase substrate; Vector Laboratories, Burlingame, CA, USA). All antibodies were applied and diluted in SuperBlock. Slides were washed three times with PBS following the application of SuperBlock, primary antibody, secondary antibody and the ABC reagent. Biotinylated secondary antibody and ABC reagents were both applied to slides for 1 h at RT. Slides were counterstained with hematoxylin and photographed at 200 × and 400 × magnifications using an Olympus BX53 microscope (Olympus, Center Valley, PA, USA).

### 2.8. Statistical Analysis

All experiments were repeated at least three times. Quantification of results was reported as means of three independent experiments plus or minus (±) the standard deviation (SD). Statistical significance was set at *p*-value less than 0.05. Statistical significance was assessed using the two-way analysis of variance analysis (ANOVA) test and the log-rank sum test. Multiple comparisons made using two-way ANOVA were corrected for by Bonferroni’s method; all *p*-values reported were therefore adjusted using this approach. Multiple comparisons were not made for the log-rank sum test, therefore this reported *p*-value was not adjusted. All statistical tests were conducted using GraphPad Prism 5 software (Microsoft, Redmond, WA, USA).

## 3. Results

### 3.1. AdUV with Wildtype E1 and Mutated E3

**Figure 1:**
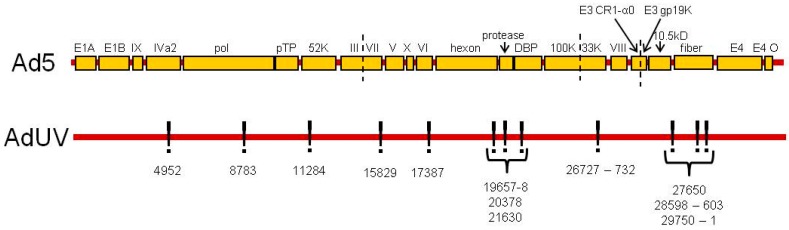
DNA mutations contained in AdUV. Map of the 12 mutations in the AdUV genome which altered their respective encoded amino acids. Ads: adenoviruses; pTP: precursor terminal protein; DBP: DNA-binding protein.

AdUV was isolated following the repeated UV irradiation of *dl*309 (Ad5), an *E1*-wildtype and *E3*-deleted Ad [42,48]. The irradiated viral progeny were then subsequently selected within cancer cells to isolate AdUV [41]. The *E1* and *E3* sequences of AdUV were shown to match the Ad5 genome, validating Ad5 as the parental strain of AdUV. Twenty-five AdUV mutations with a low error rate of sequencing (≤15%) are listed in Table 1 (GENEID# AC_000008.1). Thirteen of these 25 mutations were either located within introns or did not alter the coded amino acids sequences of their respective genes, we therefore focus upon the 12 mutations which were shown to alter their respective coded amino acid sequences in the AdUV genome relative to Ad5 (Table 1 and Figure 1).

### 3.2. Mutations That Affect Ad Packaging

There are several mutations in genes associated with virus packaging (Figure 1 and Table 1, highlighted in blue). Mutation at position 4952 changed the encoded threonine amino acid to a serine in the IVa2 gene which is associated with virus DNA packaging and the transcription of the major late Ad promoter [49,50]. AdUV was previously shown to rapidly transition from early to late gene expression [41]. The change from threonine to serine meant that AdUV lost one branched methyl group from the IVa2 polypeptide side-chain. While both threonine and serine serve very similar biochemical functions, the pivotal role of IVa2 to package the Ad genome indicates the potential relevance of this mutation. Since threonine and serine are both polar uncharged amino acids with very similar structures, we consider this mutation to be conservative. It is possible however that this mutation may have altered the tertiary or quaternary structure of IVa2 by replacing threonine with a smaller serine residue.

The Ad terminal protein or pTP was mutated at position 8783 (Table 1 and in Figure 1). The pTP peptide is the precursor form of the Ad terminal protein which matures following protease cleavage [51,52]. pTP stabilizes the origins of Ad DNA replication, promoting DNA synthesis by the formation of a pTP/pol dimer and the recruitment of the transcription factors octamer-binding protein 1 (Oct-1) and nuclear factor I (NFI) which bind pTP and pol, respectively [53,54]. In AdUV, pTP is mutated at 8783, changing an encoded arginine with a positive charge at physiological pH to a hydrophobic leucine.

A mutation at position 11284 to *52K* replaced a hydrophobic tyrosine residue with histidine, an amino acid with a positive charge at physiological pH (Table 1 and in Figure 1). The 52K protein is a DNA binding protein [55,56]. It has been shown that the positively charged amino acids at 105, 106 and 107 residues, respectively, were shown to be essential for 52K function [57], therefore, it is possible that the addition of another basic amino acid residue may enhance these functions.

In AdUV, the pV gene was mutated at two sites: 16588 (silent mutation) and 17387. The mutation at 17387 changed the endogenous glycine amino acid to an arginine residue (Table 1 and in Figure 1). Glycine has a single hydrogen atom as a side-chain, while arginine has a large, six-carbon nitrogenous side-chain with a positive charge at physiological pH. The endogenous function of pV is to form a shell surrounding the condensed Ad DNA during virion packaging onto the interior surface of the capsid during virion assembly by interaction with *pVI* and *p32* [58,59,60]. The DNA binding functions of pV has been described as histone-like in infected cells [61]. There are 16 known pV DNA binding sites, however, the DNA binding sites of pV may be altered by the interaction with pVI, pVII, or μ [62]. These pV peptide DNA binding sites were identified via PONDR-FIT, version VL-XT [63]. This mutation (17387) observed in our study replaced the 281 glycine residue with an arginine which is not located in a known pV DNA binding site [61]. Therefore, the substitution of the glycine hydrogen atom side-chain for the positively charged arginine may have generated a novel site for pV to bind the negatively charged DNA sugar-phosphate backbone with greater affinity to promote viral packaging, leading to greater progeny production and increasing the oncolytic efficacy of AdUV.

A mutation was also observed at 26727–32 which deleted six DNA nucleotides, removing two encoded alanine residues from 33K (Table 1 and in Figure 1). The 33K protein is known to have functions related to Ad DNA packaging, the expression of IIIa and pVI proteins as well as its role as an alternative RNA splicing factor [64,65]. Furthermore, mutant viruses with 33K deleted were shown to produce empty Ad capsids devoid of Ad DNA, demonstrating the requirement of 33K expression to efficiently package Ad DNA [65]. 33K has also been shown to form a dimer with IVa2 to upregulate the transactivation of the major late Ad promoter which helps to promote virion assembly and release [50,66].

The effect of these mutations may co-operate to bind and package Ad DNA more effectively and stimulate the rapid progression of AdUV from the early to late stages of Ad replication via enhanced Ad major late promoter transactivation.

### 3.3. Mutations Affecting Other Ad Structural Proteins

There were also several mutations in other virus structural proteins (Figure 1 and Table 1, highlighted in green). A single nucleotide deletion in *pIII* at 15829 resulted in a non-sense mutation which changed the encoded glycine to an alanine and also produced a premature stop codon, preventing the translation of twelve amino acids (560–571, IVSPRVLSSRTF) into the pIII peptide (Table 1). The pIII gene has also been implicated for its role to package Ad DNA [67]. Once expressed, pIII binds with four other pIII macromolecules to form the penton base, which is located at each of the 12 vertexes of the icosahedral Ad capsid [68]. Ad serotype 2 encoded pIII peptide (98.6% similarity to Ad5 by BLAST peptide analysis) was shown to be the most highly phosphorylated peptide in the Ad2 phosphatome with 12 total phosphorylation sites: one tyrosine, one threonine and 10 serine residues [69]. This truncated form of *pIII* expressed by AdUV may form smaller pIII pentamers (penton) with fewer phosphorylation sites.

Three of six mutations in hexon protein were shown to change their respective amino acid sequences (Table 1 and Figure 1). The mutations at 19657–8 changed a polar uncharged threonine into an alanine; the mutation at 20378 changed a leucine to a valine; the mutation at 21630 changed the encoded the positively charged arginine to a smaller, polar uncharged glutamine residue (Table 1 and Figure 1). Hexon is one of the principal components of the Ad capsid. Each facet of the Ad capsid contains twelve hexon trimers with hexon pentamers positioned at each vertex of the icosahedral capsid [70,71]. in vivo, hexon is highly immunogenic [72] and contains many hypervariable regions (HVRs) which can be bound by antibodies to neutralize the infectivity of Ads [73]. Hexon is known to contain nine HVRs located in the DE1 and FG1 loops between the coded 126 and 461 amino acids [73]. The mutations located at 20378 and 21630 are not contained within one of these nine HVRs. The 19657–8 mutation changed a threonine residue to an alanine at 273aa; however, this mutation is also not located within any of these nine *hexon* HVRs. Therefore, these mutations should not alter the coded amino acids of the hexon HVRs. However, their effects upon hexon immunogenicity in vivo is unknown.

One mutation in *pVIII* at 27650-1 was shown to alter the amino acid by changing the endogenous hydrophobic, cyclic proline to a polar uncharged serine residue (Table 1 and Figure 1). Proline is the only known cyclic amino acid and can form sharp turns or kinks in polypeptide structures which cannot be formed by other amino acids [74]. The capsid protein pVIII is known to interact with pV on the interior of the Ad capsid [75], which together with pVI, helps to connect the Ad capsid vertexes to the interior of capsid and ultimately to the Ad DNA genome as virions are assembled [75]. The pVIII peptide has several proline residues, from 37 to 90, which form a tight α-helical structure which interacts with hexon within the capsid [76]. There are also a number of key proline residues in pVIII which contribute to a U-bend and α-helix structures [76]. This AdUV mutation did substitute a proline for a serine at residue 160 however, this mutation did not affect these crucial alpha helical (37–90) and U-bend (63–77) proline-rich domains. Therefore, this mutation may not affect these bends and twists in pVIII. This mutation may have also brought pVIII, pV and pVI into a more open conformation or increased the affinity of the capsid for Ad DNA, leading to greater oncolysis and spread in solid tumors.

### 3.4. AdUV Displayed Greater Oncolysis and Release from A549 and Saos-2 Cells

**Figure 2:**
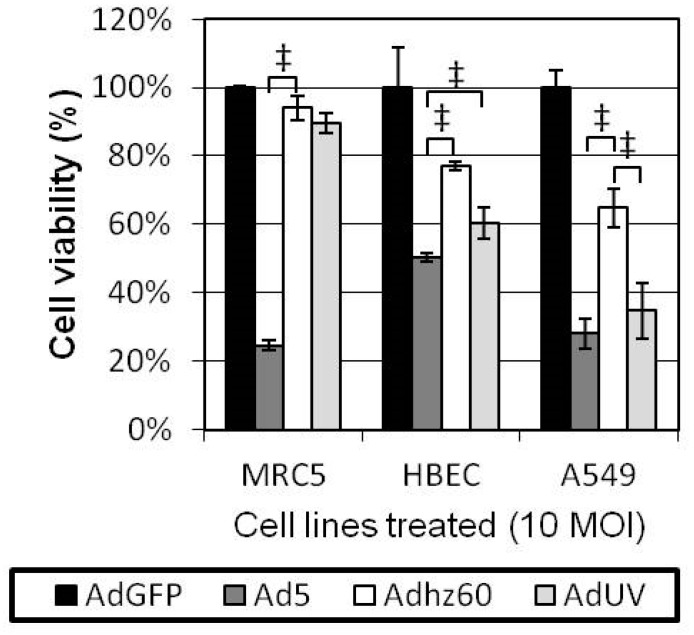
AdUV displays similar cancer selectivity to Adhz60 in A549 lung cancer cells relative to HBEC and MRC5 non-cancerous lung cells in vitro. A549 lung adenocarcinoma, MCR5 non-cancerous lung fibroblast and HBEC non-cancerous lung epithelial cells were infected with the indicated Ads at a multiplicity of infection (MOI) of 10 for three days. They were then fixed with formaldehyde and stained using crystal violet. These results were then quantified and expressed as the percentage cell viability. Data were then analyzed using two-way ANOVA with multiple comparisons corrected for by Bonferroni’s method. ‡, indicates *p*-value < 0.001.

As AdUV contains the wildtype *E1* region and thus may be less cancer selective, we therefore compared the lytic effects of AdUV upon cancer and non-cancerous cells. MRC5 non-cancerous lung fibroblasts, HBEC non-cancerous lung epithelial cells, and A549 lung cancer cells were each treated with AdUV, the non-selective Ad5, and the cancer selective *E1b*-deleted Adhz60 at an MOI of 10. Cell viabilities were determined via crystal violet staining after 72 h. Ad5 was shown to lyse MRC5 and HBEC non-cancerous lung cell lines more effectively than Adhz60 (*p*-value < 0.001) and AdUV (*p*-value < 0.001; Figure 2). While AdUV was shown to lyse significantly fewer HBEC cells than Ad5, AdUV may lyse normal lung tissue in vivo. We consider this to be unlikely as Ad5-based oncolytic Ads have not been shown to induce pulmonary toxicity clinically. AdUV, like Ad5, lysed A549 lung cancer cells more effectively than Adhz60 (*p*-value < 0.01; Figure 2). Therefore, AdUV kills lung cancer cells efficiently, indicating that these AdUV mutations may have enhanced its cancer cell lytic potential.

**Figure 3:**
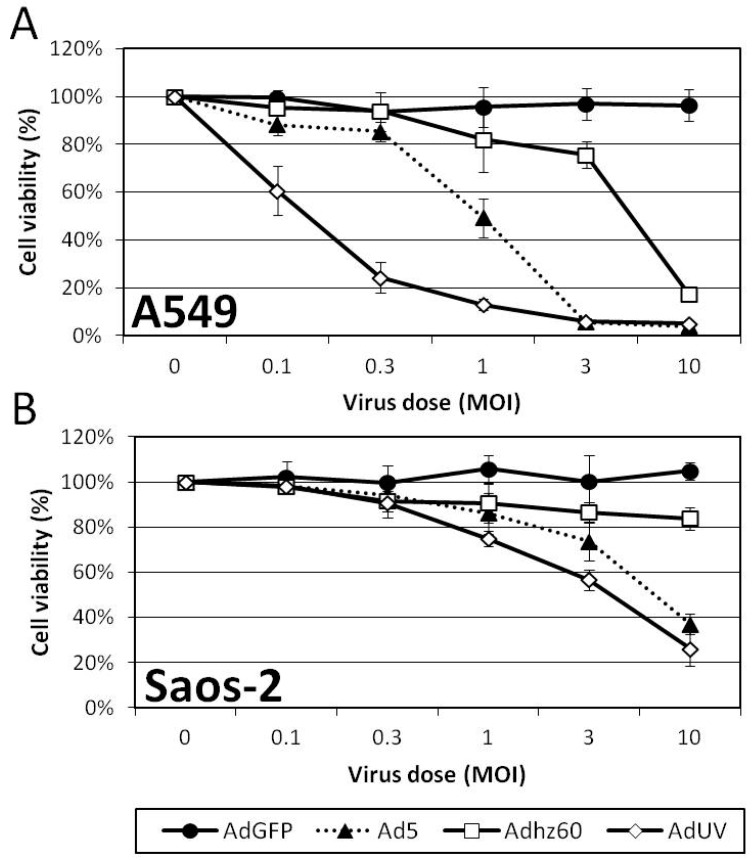
AdUV displays greater oncolysis than Ad5 and Adhz60 in A549 and Saos-2 cancer cells. (**A**) A549 and (**B**) Saos-2 cells were infected with the indicated viruses and MOIs for five days prior to crystal violet staining. The absorbances of these treated cells were normalized relative to non-treated cells.

The effect of AdUV upon A549 and Saos-2 cancer cell viability was determined by crystal violet staining following treatment with the indicated Ads for 5 days. A549 cells are known to support greater Ad replication relative to Saos-2 cells [20]. A549 cells displayed the greatest differences in cell viability at an MOI of 1; these viabilities were 91%, 40%, 67%, and 10% for AdGFP, Ad5, Adhz60, and AdUV, respectively (Figure 3A). Saos-2 cells displayed the greatest differences in cell viability at an MOI of 3, and the viabilities were 100%, 74%, 87%, 57% for AdGFP, Ad5, Adhz60, and AdUV, respectively (Figure 3B). These data indicate the enhanced cancer lysis of AdUV compared with the parental strain Ad5.

**Figure 4:**
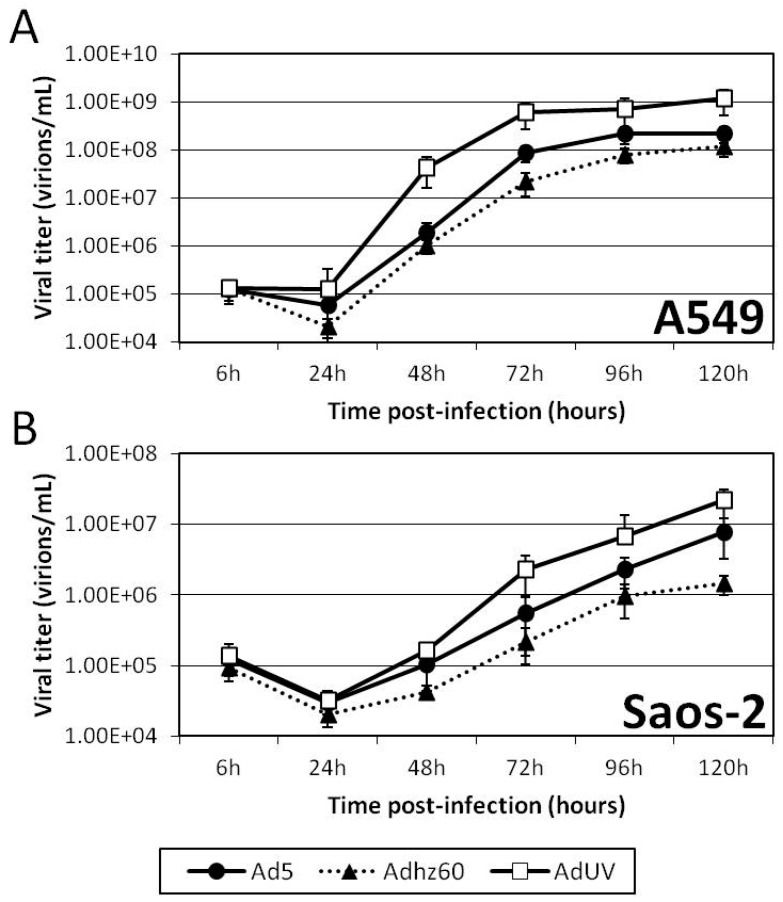
AdUV displays greater release than Ad5 and Adhz60 in A549 and Saos-2 cancer cells. (**A**) A549 and (**B**) Saos-2 cells were infected with the indicated Ads at an MOI of 1. Day 0 media samples were collected 6 h post-infection; after these samples were collected, cells were then washed with cell culture media to remove non-internalized adenovirus particles. Media samples were then collected every 24 h for five days. These samples were then titered on HEK293 cells using the median tissue culture infective dose (TCID50) method.

To study AdUV release from cancer cells, A549 and Saos-2 cancer cells were infected with AdUV, Ad5, or Adhz60 at an MOI of 1. Six hours after infection, these cells were washed with fresh culture media prior to collecting 25 µL of the cell culture media every 24 h for five days. These samples were then titered on HEK293 cells using the TCID50 approach. The titer of each Ad was shown to increase with time in both A549 and Saos-2 cancer cell lines (Figure 4A,B). AdUV displayed higher viral titers than Ad5 and Adhz60 at each time point in A549 and Saos-2 cells. At 72 h, Ad titers released from A549 cells in the medium were 3.3E8, 3.20E7, 1.09E7 for AdUV, Ad5, and Adhz60, respectively (Figure 4A), showing the titer of AdUV was 10 fold greater than Ad5 and 30 fold greater than Adhz60. AdUV also displayed higher viral titers in Saos-2 cells than Ad5 and Adhz60. At 72 h, AdUV titer was 1.35E6, while titers of Ad5 and Adhz60 titers were 4.20E5 and 1.16E5, respectively (Figure 4B). These data indicate that AdUV is released from treated cancer cells much more rapidly and efficiently than Ad5 and Adhz60.

### 3.5. AdUV Inhibits Tumor Growth in Nude Mice

**Figure 5:**
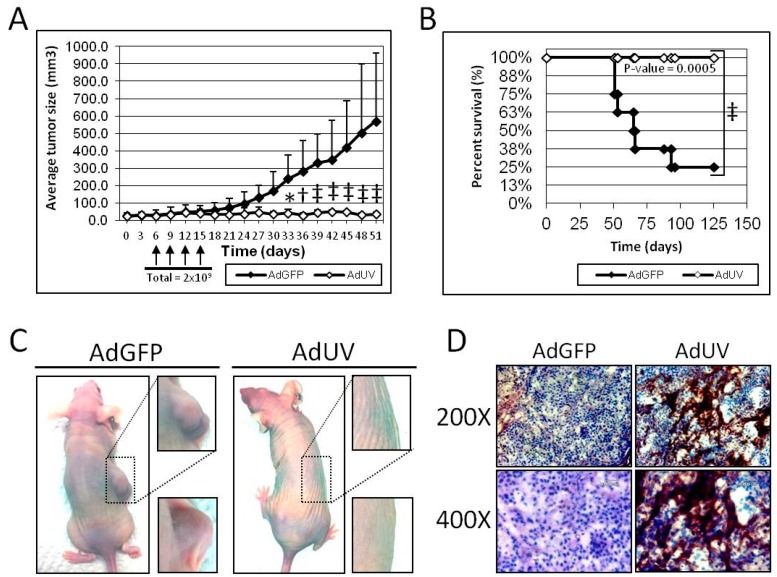
The effect of AdUV upon A549 xenograft tumor growth and nude mice survival. Xenograft tumors were formed following the subcutaneous (SC) injection of 5 × 10^6^ A549 cells into the right flank of athymic nude mice (NCr-nu/nu). (**A**) Tumors were then intratumorally (IT) injected with AdGFP or AdUV four times from day 6 until day 15 for a total dose of 2 × 10^9^ PFU; (**B**) Mice were sacrificed once tumors were greater than 1000 mm^3^ in size. Data were expressed as the percent (%) of death among each treatment groups across time; (**C**) Representative mice treated with AdGFP and AdUV were photographed at day 51. Boxes indicate the magnified (~3.2× total magnifications) right flanks of two representative mice per treatment group; (**D**) Immunohistochemistry for hexon expression in A549 tumors harvested seven days after the final injection. Slides were photographed at 200× and 400× total magnifications respectively. Differences in tumor growth were assessed by two-way ANOVA. Adjusted *p*-values were reported for multiple comparisons via Bonferroni’s method at each time-point. Differences in murine survival were determined using the Kaplan-Meier log-rank sum test. * indicates *p*-value < 0.05, † indicates *p*-value < 0.01, ‡ indicates *p*-value < 0.001.

The efficacy and safety of AdUV was further tested in vivo using an A549 subcutaneous athymic nude mouse (NCr-nu/nu) xenograft model. Once tumors grew to 30 mm^3^, mice were randomized and treated via intratumoral injection with 5 × 10^8^ plaque forming units (PFU) of AdUV or the negative control AdGFP every three days for a total of four treatments (Figure 5A). The length and width of each tumor were measured using a caliper every three days. Mice treated with AdUV exhibited significant suppression of tumor growth with 94% reduction in mean tumor volume compared with mice treated with the control AdGFP vector at day 51 (*p*-value < 0.001, Figure 5A). Furthermore, all AdUV treated mice (*n* = 7) survived until day 125 (*p*-value = 0.0005) while only two AdGFP treated mice (*n* = 8) survived to that time point (Figure 5B). Representative photographs were taken of mice treated with AdGFP or AdUV at day 51 to display the marked qualitative differences between the two treatment groups (Figure 5C). Furthermore, weight loss or lethargy was not observed following AdUV treatment. In addition, AdUV was shown to express the viral late protein, hexon, in A549 xenograft tumor sections seven days after the final viral injection, which was not detected in tumors treated with AdGFP (Figure 5D). These results indicate that AdUV can be safely used in animals to suppress tumor growth.

## 4. Discussion

The goal of this study was to investigate which genes in Ad genome may affect Ad oncolytic efficacy, and, therefore, may be modified and combined with other approaches in the development of future oncolytic Ads. The Ad early genes in AdUV, *E1a* and *E1b*, *E2*, and *E4*, remained unchanged however, some critical Ad late genes were mutated and are worthy of additional study. These AdUV mutations likely enhanced Ad-mediated cancer cell lysis and cancer selectivity. Twelve of these mutations in the AdUV genome of interest were shown to alter the encoded amino acids, five of these mutations occurred within Ad capsid structural genes (*pIII*, *Hexon*, *pVIII*; Table 1), five of these mutations occurred within genes encoding DNA binding or DNA packaging proteins (*IVa2*, *pTP*, *52K*, *pV*, *33K*; Table 1). Another two mutations, *E3A 10.5kD* and *E3 Cr1-α0*, were not discussed in detail, which may affect the receptor internalization and degradation (RID) pathway and immune cell–cell interaction, respectively. The absence of mutations in the early viral genes, *E1* and *E4*, was surprising as these genes have important roles in an array of virus–host interactions. By far, the development of cancer selective Ads has focused upon the modification of the Ad early genes by the deletion of the *E1b* genes, *E1b*-19K and *E1b*-55K, or the regulation of *E1a* gene expression using cancer-selective promoters [23,77]. However, our study has shown that the late gene-encoded structural proteins may also influence Ad replication, release, and oncolysis [41]. This hypothesis agreed with a previous report showing that a mutation to Ad5 at 8350 was associated with large plaque formation in cancer cells due to the truncation of 21 amino acids in a gene called i-leader [78].

In this report, we describe mutations in six different Ad proteins with known roles to package viral DNA during virion maturation. These Ad capsid proteins include IVa2, pTP, 33K, pV, 52K and pVIII. In particular, pV, IVa2, pVI and pVIII interact with and anchor Ad DNA onto the capsid [79]. The mutations in pVIII, pV and IVa2 are all on the interior of the capsid and have been shown to co-operate to facilitate the packaging of DNA into the Ad capsid to produce mature virions. The mutations occurring within pV, and its interaction partners IVa2 and pVIII, indicate the importance of these proteins for DNA packaging which could support the rapid release of Ads.

We previously reported that AdUV was shown to rapidly transition from early to late phase gene expression [41]. IVa2 and 33K have been shown to form a complex to stimulate the transcription of the major late Ad promoter [50,66], it is possible these *IVa2* and *33K* mutations found in AdUV may have increased the formation of this complex to induce early to late phase transition during Ad replication. Furthermore, there exists indirect evidence that IVa2 and 33K can also form a complex with DNA-binding protein (DBP) on one of the Ad capsid vertexes facilitating Ad DNA packaging [50]. These mutations acting together may be associated with rapid AdUV virus assembly and release.

Our results have also shown that AdUV can be safely used in animals and suppress tumor growth. AdUV is an *E1b*-wildtype Ad. In early oncolytic studies in the 1950s, wildtype Ads were the first viruses used in clinical trials [80]. However, wildtype Ads are no longer used in clinical studies because of safety concerns and their limited cancer therapeutic efficacy. For this reason, AdUV, based on wildtype Ad5, is unlikely used in clinical studies in the future. Therefore, our intention was to perform limited animal experiments to investigate whether the mutations generated in AdUV may be toxic to animals. To further study these mutations, each mutation should be individually introduced into a well-characterized Ad vector background to understand their impact upon Ad replication and oncolytic therapy. Then, some AdUV mutations may be selected for integration into oncolytic Ads that have strong, well-characterized cancer selectivity and are able to stimulate strong anti-tumoral immune responses.

The expression of *E1b* by AdUV also likely increased its oncolytic potential. Several other *E1b*-wildtype oncolytic vectors have been described with increased cancer therapeutic efficacy containing other modes of cancer selectivity such as cancer selective promoters, or partial *E1a* deletions [81,82]. Even though *E1b* expression is not essential for Ad replication in cancer cells [20], *E1b* expression may enhance the efficiency of Ad replication in cancer cells, likely via cyclin E induction [20] and autophagy activation [83]. We have previously shown that E1B55K can enhance viral *E1a* expression and is involved in the induction of cyclin E which is required for efficient Ad replication [9,18,19,20,21,22,23]. Our recently published studies have also shown that *E1b* upregulates autophagy [41]. Autophagy is known to degrade intracellular components, organelles and long-lived proteins, to produce amino and fatty acids for cell survival [84,85,86]. It appears that *E1b* expression supports autophagy induction by Ads to promote virus replication and cancer cell lysis by generating nutrients for building viral particles [35,83]. Therefore, the oncolytic replication of AdUV may have benefited from its *E1b*-wildtype status.

These findings taken together provide additional insight into the construction of oncolytic Ads. It appears that *E1b* expression and mutations in the Ad late proteins, especially those associated with genome packaging, may augment Ad release and spread within tumor tissue, leading to enhanced oncolytic therapy efficacy. One of the limitations of this study is that we do not know the specific effects of each of these mutations upon oncolytic replication at this time; it is possible that several of these mutations may cooperate to enhance oncolytic virotherapy efficacy.

## Figures and Tables

**Table 1:** Table of AdUV mutations, their location, and changes in their amino acid sequences. Conservative mutations are defined as changes to the encoded amino acids which have led to similar biochemical properties. We were interested in those non-conservative mutations in which DNA mutations altered the biochemical properties (charge, hydrophobicity and size) of these coded amino acids. Within the table, blue rows indicate mutations to DNA binding and packaging genes, green indicate mutations to Ad structural genes and white indicates mutations to genes which are not related to Ad structure or DNA binding. These mutations were shown to have a low DNA sequencing error. The total Ad genome is 36 kilobases (kb). aa: amino acid; DN: double nucleotide; SN: single nucleotide.

Site	Gene	Mutation	aa Changed?	Non-Conservative Mutation?
4952	*IVa2*	G to C	T to S	No, T and S are both polar uncharged aa
8783	*pTP*	G to A	R to L	Yes, R is basic (+) and L is hydrophobic
11284	*52K*	T to C	Y to H	Yes, Y is hydrophobic and H is a positivity charged aa
12728	Intron	SN deletion	No	No
15829	*pIII*	SN deletion, non-sense mutation	G to A and lost 13 aa: IVSPRVLSSRTF	Yes, lost 13 aa
16588	*pV*	G to A	No	No
17387	*pV*	G to C	G to R	Yes, G is hydrophobic and R is basic (+)
18755	*pVI*	SN deletion	No, targeted only the last codon. Did not remove the codon stop	No
19483	*Hexon*	T to A	No	No
19513	*Hexon*	T to A	No	No
19657–8	*Hexon*	DN substitution, GA to AG	AT to AA	Yes, T is polar uncharged and A is hydrophobic
20378	*Hexon*	T to C	L to V	No, both L and V are hydrophobic
21163	*Hexon*	C to T	No	No
21630	*Hexon*	G to A	R to Q	Yes, R is basic (+) and Q is polar uncharged
25995	*100K*	A to T	No	No
26561	Intron	G to A	No	No
26727–32	*33K*	Six-nucleotide deletion	Lost 2 aa A and A	Yes, lost 2 aa
27161	Intron	C to T	No	No
27314	*pVIII*	C to A	No	No
27339	*pVIII*	T to C	No	No
27650–1	*pVIII*	DN substitution TC to CT	RP to RS	Yes, P is cyclic and S is polar uncharged
28120	Intron	T to C	No	No
28596–601	*E3 CR1-α0*	Six-nucleotide deletion	Lost 2 aa I and G	Yes, lost 2 aa
29750	*E3A* 10.5kD	T to G	M to R	Yes, M is hydrophobic and R is basic (+)
35776	Intron	A to C	No	No
